# Vaccine hesitancy among general practitioners in Southern France and their reluctant trust in the health authorities

**DOI:** 10.1080/17482631.2020.1757336

**Published:** 2020-05-13

**Authors:** Rose Jane Isobel Wilson, Chantal Vergélys, Jeremy Ward, Patrick Peretti-Watel, Pierre Verger

**Affiliations:** a ORS PACA, Observatoire Régional de la Santé Provence-Alpes-Côte d’Azur, Marseille, France; b Aix Marseille Univ, IRD, AP-HM, SSA, VITROME, Marseille, France; c IHU-Méditerranée Infection, Marseille, France; d UMR 8236 (LIED), Université Paris Diderot, Paris, France

**Keywords:** France, general practitioners, in-depth interviews, socially constructed knowledge, trust, vaccine hesitancy

## Abstract

**Purpose: **Vaccine hesitancy is common in France, including among general practitioners (GPs).  We aimed to understand vaccine hesitant GPs’ views towards vaccines. **Method**: We conducted in-depth interviews that were thematically analysed. **Result:** We found that, facilitated by health scandals and vaccine controversies—that according to participants were not effectively handled by health authorities—the implicit contract existing between health authorities and GPs has been ruptured. This contract implies that health authorities support GPs in making vaccine recommendations by addressing GPs’ own concerns, providing them with adequate and up-to-date information and advice, and involving them in vaccine decision-making. In turn, GPs encourage vaccination to reach vaccine coverage targets. **Conclusion:** The rupture of this implicit contract has led to a breach in trust in the health authorities and the vaccines that they recommend.

## Abstract (fr)

**Objectif**: L’hésitation vaccinale est fréquente en France, y compris chez les médecins généralistes. Nous avons cherché à comprendre le point de vue des médecins généralistes expriment des incertitudes sur les vaccins. **Méthode**: Nous avons réalisé des entretiens approfondis qui ont fait l’objet d’une analyse thématique. **Résultats**: Nous avons trouvé que, favorisée en partie par crises sanitaires et des controverses vaccinales - qui selon les participants ont été mal gérées par les autorités sanitaires - le contrat implicite entre les autorités de santé et les médecins généralistes a été rompu. Ce contrat implique que les autorités aident les médecins généralistes à appliquer les recommandations vaccinales, en répondant aux inquiétudes des médecins généralistes; en leur fournissant des informations et des conseils adéquats et à jour; et en les impliquant dans la prise de décision en matiére de vaccins. À leur tour, ceux-ci sont vacciner afin d’atteindre les objectifs de couverture vaccinale. **Conclusion**: La rupture de ce contrat implicite a suscité une rupture de confiance envers les autorités sanitaires et des vaccins qu’elles recommandent.

## Background

For the majority of those in high and middle-income countries, vaccination is part of an established health care routine. However, many countries are experiencing a reduction in vaccine confidence. The 67-country survey conducted by Larson et al. in 2016 found that the global average of safety and effectiveness-related vaccine scepticism was 13% and 9% respectively. Respondents from France (45%), Bosnia & Herzegovina (38%), and Japan (31%) reported the highest rates of safety-related scepticism, and Bosnia & Herzegovina (27%), Russia (20%), and Italy (19%) had the highest rates of effectiveness-related scepticism. In general, the European region had lower confidence in the safety of vaccines than other world regions. Moreover, this region accounted for seven of the ten countries with the lowest levels of safety-based confidence issues, including France, Greece, Slovenia, and Italy. More recently, vaccine confidence has significantly decreased in Poland, Sweden, Finland and Belgium. (Larson, de Figueiredo, et al., 2018).

In France unfavourable opinions towards vaccination have increased over the past 20 years (Ward et al., 2019). As stated above, in 2016 France was identified as the country with the lowest confidence in the safety of vaccines (Larson et al., 2016). Today it remains one of the countries with the lowest confidence in vaccine safety, even if overall confidence in vaccine safety has increased (Larson, Clarke, et al., 2018). This has contributed to low vaccine coverage rates; acceptance of two doses of the childhood the measles vaccine, for example, only reached 80% in 2016 (Rey et al., 2018) (compared to the target of 95% coverage (WHO, 2017)). This led to more than 2,500 cases of measles from January to May 2018, including three deaths and high rates of hospitalization (22%) (Santé publique France, 2019a). Given this situation, in 2017 the Ministry of Health (MoH) extended mandatory childhood vaccines from three (diphtheria, tetanus, poliomyelitis) to 11 vaccines (including hepatitis B, meningitis C, and measles, mumps and rubella (MMR). These vaccines are now required by law, for children to be admitted to creches and schools. Other vaccines (e.g., against papilloma virus remain recommended).

Distrust in some vaccines or the vaccination process can lead to vaccine hesitancy; being uncertain about specific vaccines or vaccination in general. This is not only experienced by patients but also some health care professionals (HCPs), including general practitioners (GPs). While GPs generally hold higher levels of vaccine confidence than the public, 36% of GPs surveyed in the Czech Republic and 25% in Slovakia did not agree that the MMR vaccine is safe and 29% and 19% (respectively) did not believe it was important. The majority of GPs surveyed in these countries reported that they are not likely to recommend the seasonal influenza vaccine (Larson, de Figueiredo, et al., 2018). In France vaccination is mainly recommended and administered by GPs, who have a pivotal role in vaccinating the population. A quantitative survey of GPs in France found that while most (80%) stated being very favourable to vaccination in general, almost a quarter were sceptical about the value of some officially recommended vaccines. Overall, moderate to severe vaccine hesitancy affected one in eight GPs (Verger et al., 2016). This finding is important considering that patients generally trust their GP as an information source (Wilson, 2017), and that there is a correlation between GP vaccine confidence and confidence among the public (Larson, Clarke, et al., 2018). This means that GP vaccine hesitancy could exacerbate patient concerns and contribute to insufficient vaccine coverage. It is also a concerning finding in France because recently (2018) GPs were given the responsibility to provide eight additional mandatory vaccines for children on top of the three previously mandatory vaccines (Ward, Colgrove et al., 2018).

It may come as a surprise that GPs could be vaccine hesitant; they are normally expected to trust the health care processes and technologies advised by health care authorities and to follow the latter’s advice by recommending vaccination. However, while GPs possess vaccination expertise beyond that of most patients, like patients, they are not part of the institutional process that leads to their production and recommendation. To get access to information and guidelines about vaccination and infectious diseases, GPs must consult the MoH’s website; indeed, a website specifically focusing on vaccination and dedicated to health care professionals has only existed since 2018. This means that they must trust that the data at the foundation of these recommendations are reliable and that experts and policymakers are making correct decisions.

GPs’ paradoxical position—at the interface of both the public and the expert systems in charge of vaccination, placing them simultaneously within and outside of this system-is also evident in the individualist approach of healthcare introduced through neoliberal reforms in the West in the 1990s (Lindberg & Lundgren, 2019). Healthcare was personalized to meet the needs and desires of individuals, which of course has very positive aspects. However the ethic of patient choice exists in a context where expert knowledge is not available for all, and often decisions related to healthcare take a lot of time and energy to make. This shifts an enormous burden onto the patient and healthcare professional under the guise of the “gift” of choice. In this way, ideals of patient choice can clash with expectations of support and advice-giving and lead to confusion and distrust. Therefore, despite the ideology that introducing patient choice into healthcare empowers patients and makes space for their desires, it in fact alters healthcare practices in ways that do not necessarily fit well with the intricacies of different people’s healthcare needs (Mol, 2008). The ideal is also full of contradictions; while there is a constant push for active decision-making, this is coupled with a wish for patients and healthcare professionals to passively comply with medical advice. The desire for patient choice thus can lead to a tension between the emphasis on maintaining health at the population level, and citizen’s individual rights to peruse their own health (Poltorak, 2007).

In France no physical contract exists between GPs and the MoH; GPs are effectively self-employed but paid by patients usually through the national health insurance fund, which, together with members of the MoH and GP trade unions, negotiates consultation fees. However, with regards to vaccination, an *implicit* contract exists between health authorities and GPs, which states that authorities define vaccine strategies (including official vaccination recommendations), and give advice and support to GPs to recommend and administer vaccines. GPs in turn are responsible for applying the vaccination strategy.

Even if an explicit contract does not exist, the trust required for such an exchange (or implicit contract) is an expectation of competence, predictability and impartiality on the part of those involved (Zaheer et al., 1998). Various studies have shown that public trust in the government is linked to a significant positive association in vaccine uptake (Lee et al., 2016), (Fu et al., 2017). Attitudes towards vaccination often reflect critical engagements (or disengagement) with local and national political histories, and the legacy of particular interactions between populations and institutions of the state, science, and the media (Leach & Fairhead, 2007). The internet for example, is one of the primary sources of information for people’s health decisions (Ward et al., 2015). Social media platforms especially have become an arena for promoting both anti- and pro-vaccination content; one survey found that half of parents with young children were exposed to negative vaccination messages on such platforms (Royal Society for Public Health, 2018). Interestingly Facebook labels vaccination-related content as political, which has angered some scientists as it “perpetuates the false idea that there is even a debate to be had” (Large, 2019). However, vaccination, as an intervention on the body provided by the state, is highly political. The worry about receiving too many vaccines for example, could echo everyday experiences and concerns with unpredictable and complex government and technical systems (Biss, 2015).

Trusting health authorities may thus be especially difficult for GPs in France in a context of repeated, high-profile health care scandals and the health authorities’ problematic handling of them, as well as GPs’ exclusion from the management of various epidemics over the past few decades. A scandal surrounding contaminated blood for example, occurred in the 1990s after it was found that the National Centre of Blood Transfusion knowingly distributed products contaminated with HIV (Ingram, 1999). More recently it was discovered that the drug Médiator® [benfluorex], licenced for hyperlipidaemia and diabetes, caused between 500 and 2000 deaths in France during its 33 years on the market (Mullard, 2011).

In terms of vaccination, in 1998 the health minister withdrew hepatitis B vaccination in schools, pending an investigation into the possibility that it could cause multiple sclerosis in adolescents. The vaccine was not re-instated in schools, and public concerns remain, despite evidence that there is no link to multiple sclerosis (Langer-Gould et al., 2014). There have also been concerns about the efficacy and safety of the HPV vaccine (possibly partly caused by the authorities’ rapid approval of the vaccine which surprised GPs and preceded the distribution of information about HPV (Lefèvre et al., 2017)), and especially around the influenza A(H1N1) (swine flu) vaccine. In 2009 there was a heated controversy over the cost of the government’s large influenza vaccination campaign, the lack of transparency in the purchasing of the vaccines, perceived alarmist communication by public health authorities, conflicts of interest on the part of some MoH advisers, and the safety of the vaccine (Ward, Colgrove, et al., 2018). The campaign failed, with only eight percent of the population vaccinating (Guthmann et al., 2010). Additionally, like the hepatitis B controversy, GPs felt excluded from the organization of the campaign, as the MoH advised that people should be vaccinated by nurses or medical students in vaccination centres (Schwarzinger et al., 2010). Confidence and perceived safety in the seasonal influenza vaccine in France currently ranks 28th and 21st respectively out of all 28 EU member states (Larson, Clarke, et al., 2018). After the controversies surrounding the hepatitis B vaccine and the influenza vaccine campaign, in 2016 the health authorities launched a national debate involving citizens, health professionals, and experts; among experts, mandatory vaccination emerged as a solution to the situation, but remained controversial among citizens and health professionals (Ward, Cafiero, et al., 2018).

To our knowledge, only two studies have specifically examined HCPs’ trust in the health care system and their likelihood to recommend vaccination (McPhillips et al., 2001; Raude et al., 2016). These studies indicate that combined distrust in the health care system, science and the government is associated with being less likely to recommend vaccination. Following the quantitative study mentioned above (Verger et al., 2016), this paper aims to better understand the context of vaccination in France and how it has contributed to vaccine hesitancy among GPs.

## Methods

### Study context

In 2014, through a national cross-sectional survey of 1712 GPs in France, we found that moderate to severe vaccine hesitancy affected one in eight GPs (Verger et al., 2016). Between November 2016 and April 2017 we conducted a qualitative study to better understand vaccination practices and concerns among vaccine hesitant GPs.

### Setting

Participants for this qualitative research were recruited in the South of France from both rural and urban areas in two regions; Provence-Alpes-Côte d’Azur (PACA) (in South-eastern France) and Occitanie (which lies to the West of PACA),^1^ which both had relatively low vaccine coverage rates compared to the rest of France. The national average coverage rates for 24-month-olds of three doses of the hepatitis B vaccine, two doses of the MMR vaccine and three doses of the recommended HPV vaccine by age 16 is 90%, 80% and 21% respectively (Santé publique France, 2019b).

However, in PACA in 2016^2^ coverage for 24-month-olds was 79% for three doses of the hepatitis B and 71% for two doses of the MMR vaccine. Coverage was 15% for three doses of the recommended HPV vaccine by age 16 (Santé publique France, 2018a). In Occitanie in 2016 coverage for 24-month-olds was 85% for three doses of the hepatitis B was and 76% for two doses of the MMR vaccine. Coverage was 17% for three doses of the HPV vaccine by age 16 (Santé publique France, 2018b).

### Study design

GPs were identified through the Yellow Pages online (an online business directory) and contacted by telephone by CV. Those who agreed to participate were included if: 1) they practiced in the PACA *or* Occitanie regions; 2) were likely to be vaccine hesitant at least to some degree. This was determined by their answers to two telephone questions: “Do you have doubts about the benefits of any vaccines recommended by health authorities?” (possible answers: yes/no/don’t know); “Do you think that any vaccines in the vaccination schedule may be responsible for serious side-effects apart from complications relating to possible allergies?” (possible answers: not at all likely/unlikely/likely/very likely/don’t know). GPs were deemed vaccine hesitant if they answered “yes” or “don’t know” to the first question or “unlikely”, “likely” “very “likely, or “don’t know” to the second question.

Included GPs then participated in a two-stage approach. Firstly, they participated in a telephone questionnaire conducted by CV that asked about specific vaccines for which coverage is suboptimal (adapted from the one used for the 2014 quantitative survey mentioned above (Verger et al., 2016)) (Appendix B). Secondly, to elucidate GPs’ answers to the telephone questionnaire, they participated in face-to-face semi-structured interviews conducted by CV. The interview questions were based on those of the telephone questionnaire but were adapted to be suitable for in-depth interviews (i.e., allowing for longer, more nuanced answers and follow-up questions). A specific interview guide was written for each GP based on their answers to the telephone questions. The interviews aimed to elucidate GPs’ views about the benefits and risks of certain vaccines and their components; whether GPs follow the vaccination schedule; how they approach vaccine hesitant patients; and their views towards vaccination information provided by the MoH and health authorities. The face-to-face interviews took place in the GPs’ practices by appointment and were audio recorded and transcribed verbatim. All participants signed an informed consent form agreeing to their participation and that their interview would be audio-recorded.

### Participants

We identified 218 GPs. Among these, 16 could not be contacted; 100 administrative staff declined on behalf of GPs; 38 declined due to not having time or not participating in studies in general and thirty-six declined due to other reasons. Twenty-eight GPs answered the two inclusion/exclusion questions and nine were excluded as they were deemed not vaccine hesitant. Nineteen GPs were thus included in the study (and participated in the full telephone questionnaire and face-to-face interview). Interviews lasted on average 28 minutes (the shortest lasting 17 minutes and the longest 73 minutes).

Three GPs were aged 36–45; six were 46–55; eight were 56–65; and two were over 65. Eleven women and eight male GPs participated. Eleven GPs worked in a group practice and the remaining eight in their own practice. These age and gender demographics are similar to those of the general population of the PACA and Occitanie regions (Collange et al., 2018).

### Data analysis

A thematic analysis was conducted by RW (the first author of this article), working closely with CV and alongside regular discussions with the other co-authors to identify and analyse patterns (themes) from the data (Braun & Clarke, 2014). Thematic analysis was used as a “contextualist” method, characterized by constructionism; we did not seek to focus only on motivation or individual psychologies, but to theorize on the socio-cultural contexts and structural conditions that enable individual accounts (Braun et al., 2019, pp. 1–18). Conducted through a critical realist perspective, the analysis also acknowledged the ways that individuals perceive and make meaning of their experiences, and in turn, the ways the broader social context impinges on these meanings, whilst retaining a focus on the limits of “reality” (there is no singular, objective truth, instead, there is a multiplicity of interrelated and subjective understandings (Taylor & Ussher, 2001)).

As all researchers, we approached the fieldwork and analysis with pre-existing positions and ideas. Throughout the study, efforts were made to maintain self-reflexivity and an awareness of the subjective nature of the data collection and analysis. This was achieved by being aware of any assumptions being made about what participants were saying, so as not to impose pre-defined theories onto their narratives. We instead aimed to use theory to highlight the views and concerns expressed by the participants, and present them in a coherent way that we hoped would reflect their meaning.

All interview transcripts were imported into NVivo11; a qualitative data organization package. The transcripts were read several times then organized and coded into text segments with the use of a coding framework. As the study began with some key questions regarding trust in vaccination and health authorities, the coding framework was formulated both deductively (through pre-established concepts guiding the research questions), and inductively (based on salient and recurrent themes identified in the data). All transcripts were then re-read and any relevant missed text added to codes, and new codes added if necessary. Participant quotes in this article were translated from French by RW. Italicized text in quotations denote the interviewer’s questions or comments. Data was anonymized and stored according to data protection laws. All participant names used are pseudonyms.

## Results

In this section we present the results according to the following overarching themes identified in the analysis;

- GPs’ reluctant trust in the health authorities in France—This theme relates to the reluctant trust GPs had to cultivate for health authorities and their advice—despite the perceived impartiality of the latter, as well as various health scandals—in order to carry out their work.
- GPs’ adoption of socially constructed knowledge—Participants often relied on socially constructed knowledge relating to their own experiences, as well as those of colleagues to inform their views of various vaccines and whether they recommended them to patients.
- and The tensions between vaccination promotion and patient choice—A number of participants eluded to vaccine recommendations, and especially mandatory vaccination as clashing with patient choice, and possibly endangering the trusting relationship between the GP and their patient, as well as restricting their capacity for exercising professional judgement

### Reluctant trust

This theme has two sub-themes: 1) “Ignored and unsupported” relating to how GPs were not sufficiently informed or supported by the health authorities in navigating their difficult position at the interface of various unfolding healthcare incidents and the patient concerns that came with them, and 2) “The need to trust to an extent” reporting on participants’ stated need to trust in the authorities at least to an extent and not to “dig too deep” into the various incidents if they wanted to avoid paralysis in their work.

#### Ignored and unsupported

It’s complicated because you never really know about the impartiality [of authorities], in relation to firms or labs, it’s always complicated … there have been lots of previous incidents that make you wonder sometimes (Dr. Thomas).

A number of participants mentioned “previous incidents” and how they contributed to their concerns about specific vaccines; Dr. Amidane spoke about the withdrawal of hepatitis B vaccine administration in schools and said that since then, even though studies have shown that there are no severe side effects associated with the vaccine, people who experienced supposed complications were awarded compensation, so according to him “the juridical system recognises it’s complicated, even if the studies do not”. Due to the lack of clarity and communication on the part of the MoH regarding its vaccination policy, Dr. Amidane was led to make his own deductions about the vaccine. He said he never recommended the booster vaccine and believed it to be “useless”. This sentiment was reflected by other GPs:

There were lots of campaigns that raised doubts … with hepatitis B … in the end it was not clear if there were side effects and … they left it to the attending physician to enlighten [patients] … so that scalded us … I don’t think the vaccination policy has been sufficiently clarified … even health professionals did not know where we were … There’s a solitude of general practitioners in front of their patient … when people ask questions … either we tell them there are no risks and then unfortunately you experience a vaccine side effect, or we warn them against vaccination and the person ends up with hepatitis (Dr. Marie).

We have had totally corrupt recommendations from the HAS [the High Authority for Health], with people in the committees who have relations with laboratories, so we can always be critical, you must be able to be objective … I’ve gone through the *Médiator* story, I prescribed it for diabetics, we were a bit misguided (Dr. Moreau).

The GPs were at the interface of the unfolding of such issues, and the patient concerns that came with them. However, they had to manage this position without support from health authorities:

We’re occupied by our jobs and then you also have to read advanced studies? I simply don’t have the capacity as a doctor to answer questions like that [about adjuvants] (Dr. Fournier).

I trust but … there are people who are sceptical but who are not necessarily against vaccination, but if we get conflicting information, or the health authorities don’t … give clear and accurate information, people will always say “ah yes I heard that, but what actually happened was … ” [The authorities] could help us (Dr. Thomas).

GPs thus felt ignored, unsupported and even exploited by the authorities. As a result, they faced continued uncertainties about what information and advice to trust and how to approach various vaccination discussions with patients.

#### The need to trust to an extent

Despite this lack of clear information and support, the uncertainties they faced, and their awareness that they were not experts in immunology, GPs were still expected to recommend vaccination, reassure vaccine hesitant patients, and achieve high vaccination rates. They thus had to rely on vaccine experts to implement the safest and most effective guidelines:

The information I have is … limited because … it comes from an official source … we have a right to be suspicious … I’m not a scientist and I don’t read all the articles … I have to trust someone, but I don’t know … I thought of the history of contaminated blood … we had to draw conclusions … but at the same time when I see what happens on the political scene … I could be part of a scandal … so [I] ‘mostly trust’ … I hang onto something (Dr. Duval).

Coupled with their obligation to vaccinate (and feeling responsible for any ensuing adverse effects), participants thus had to “hang onto something”, (despite being “sceptical or “suspicious”): They had no choice but to trust the authorities and vaccination to an extent:

*You said you ‘mostly do not trust’ the Ministry of Health to give you reliable information on the benefits and risks of vaccines …* (Silence) they don’t react … they don’t act in relation to health, there are questions of money … There are things that come into play … other than health itself … it may be a bit ambiguous but we want to trust them anyway (Dr. Andre).

Similarly, Dr. Andre stated: “If we ask ourselves questions about everything, we’ll do nothing, we’ll prescribe nothing”. Dr. Marie expressed: “I’m not trying to dig deeper because somewhere I trust and if I dig too much … inevitably I will find something”. Vaccine hesitant GPs were thus aware that if they did not have a level of trust, and were to seriously contemplate some vaccines, a paralysis of action could ensue:

If I start not trusting … in the things that are said to me, what am I going to do? So yes, I have trust (Dr. Michel).

Trust is … it’s difficult to work without it, so it’s a priori and then sometimes we expect to be disappointed … there have already been scandals, there have already been conflicts of interest (Dr. Morel).

In summary, this theme highlights the distrust some GPs have in the health authorities following their handling of various health scandals and controversies; lack of information and support in managing patients’ concerns; and GPs’ being left out of vaccine policy-making. In order to deal with these difficulties, some participants bracketed out issues and uncertainty that they could not fully resolve, creating an illusion that issues were partially resolved, thus allowing them to *reluctantly trust* in the authorities and certain vaccines, and to continue to practice (cf. Giddens, 1991). As explored in the following section, this was aided by drawing upon additional sources of support.

### The adoption of socially constructed knowledge

This theme relates to the informal sources of vaccine information that GPs refer to, and has two sub-themes: “Unofficial sources of information” relating to the socially constructed vaccine knowledge arising from non-government and non-academic sources, and “The influence of positive and negative personal experiences on vaccine perceptions and recommendations” relating to how participants tailored their recommendations on personal vaccine experiences.

#### Unofficial sources of information

When asked why she recommended the MMR catch-up vaccine for adolescents, Dr. Dubois—who in the telephone questionnaire, said she mostly trusted advice provided by the MoH and health agencies—responded,

It’s in the recommendations! … I apply the recommendations … I have the vaccination calendar^3^ … that I systematically refer to when I have doubts … I don’t ask questions! I use the calendar … that’s my base reference (Dr. Dubois).

However, when GPs were not confident in or satisfied by official information or guidelines from the MoH, some diverged slightly from the official recommendations and turned to unofficial scientific information and advice, such as from the journal *Prescrire*: an independent medical journal that is often critical of official health care recommendations (Prescrire, 2019). Dr. Morel reported that she trusted independent reviews, tried to have her own data, and synthesize it herself. Four other participants also mentioned that they often referred to recommendations made in *Prescrire.*

Over half of the participants however, indicated that while they occasionally read vaccination studies, they usually did not have time to do this. Thus, mediated by organizational demands, and distrust in health authorities, these GPs diverged further from official vaccination guidelines. They did not read vaccination reviews at all but took alternative, more social routes to acquiring what they thought was the best evidence, from sources that were easily accessible and that they saw as competent and trustworthy, including colleagues,

I form my own opinion and compare it with colleagues on the ground … it’s much more beneficial than listening to the recommendations. I trust doctors on the ground, but not administrative staff … Look what happened with H1N1 [swine flu] … it was a monumental fiasco, they wanted to vaccinate … against the advice of doctors … I don’t usually listen, well … I form my own opinion and I think it’s much more beneficial to listen to colleagues who are on the ground than listening to the recommendations that are sometimes irrelevant (Dr. Martin).

I’m not convinced that the systematic vaccination against meningococcus C in 12-month-olds is necessary … I discussed it with paediatricians who told me “we don’t do it systematically, we just vaccinate children who go to crèches … ”, so I went with that advice (Dr. Amidane).

#### The influence of positive and negative personal experiences on vaccine perceptions and recommendations

Eight participants explicitly stated that they preferred to draw on experience-based knowledge relating to vaccines and diseases rather than official guidelines. Negative experiences with patients who suffered supposed vaccine side effects with a high emotional charge (for both patient and GP) affected how some GPs recommended vaccination or certain vaccines:

I tried to vaccinate a child whose mother was really against vaccines. I persuaded her but unfortunately afterwards the girl was hospitalised with pneumopathy … that moderates your position! … the mother made the correlation … [but] it’s challenging … *It undermines your confidence in vaccines?* Yes totally, to see that when we search, there’s no answer. I now try more to gauge the risk-benefits … it shakes you up (Dr. Laurent).

A few years ago [I vaccinated] an infant and 15 days later the infant died … the mother blamed the vaccine because she had read …—I don’t know if it was televised—… that this vaccine could create a risk … I remember very well … I insisted on the vaccine, and the child is dead … I don’t think it’s related to the vaccine but she was sure … it wasn’t mandatory but I insisted on doing it, that’s maybe why I’m more reluctant [now] … I insisted and the child died … so … now … I don’t insist or push for any non-compulsory vaccines … I leave the choice to my patients (Dr. Marie).

Such personal experiences were even sometimes drawn upon in discussions with patients. Dr. Duval tried to “motivate” his patients to vaccinate by relaying an emotive anecdote about a young woman suffering from cervical cancer with her two children watching from the end of her bed. Conversely, Dr. Bernard used his negative personal experience to discourage HPV vaccination among patients. He was asked how he responded when patients asked why they did not have to receive the HPV vaccine (according to his advice). Dr. Bernard stated:

I answer that I had a personal problem … one of my children had a side effect following vaccination. … I play my role as a doctor, so I have to offer [the vaccine] … but I’m reluctant to do it (Dr. Bernard).

In summary, nearly half of the participants stated that they preferred to draw on experience-based knowledge relating to vaccination rather than official guidelines. These informal forms of knowledge are collectively reinforced and come together to form a type of internal, or tacit knowledge, resulting in “socially constructed knowledge in practice” (Gabbay & Le May, 2004). This tacit knowledge was illustrated when even participants who spoke about the risks of disease and stated that research had proven vaccine efficacy, often “proved” this with personal and highly emotive narratives, rather than citing scientific studies: “I’ve been personally impacted by measles in my family … that’s the main reason I’m for catch-up [MMR] vaccination” (Dr. Bernard). Experiences with patients, as well as even more personal experiences (such as those with a family member) thus influenced participants’ vaccination recommendations and sometimes the direct advice they gave to patients.

In the following section we further analyse the tensions arising for vaccine hesitant GPs in vaccine discussions with patients.

### Tensions between vaccination promotion and patient choice

Health authorities aim to sustain high vaccine coverage rates in order to maintain “herd” immunity against vaccine-preventable diseases through an aim for compliance with vaccination policy throughout the population. This requires GPs to recommend vaccines, some of which are mandatory. However, this approach clashes with a simultaneous rhetoric of autonomy and patient choice in health care settings in the West, as well as (as evident in this study), GPs’ commitment to patients’ interests by taking their preferences into account. As such, the goals and values of GPs in their relations with their patients did not necessarily coincide with those of public health:

The role of the doctor is not to order … but to encourage patients to vaccinate. I follow patients’ requests; I think being too rigid would cause some patients to refuse vaccination. The relation with the patients is very much a relation of trust, so it is also necessary that the patient feels listened to, heard (Dr. Durand).

I went to meetings on vaccinations where … we were given (vaccination) cards- “here, you can argue like this … ” It’s theoretical, in practice it doesn’t work because the dialogue isn’t there … I sometimes find the pro-vaccine arguments a bit unrealistic … it doesn’t have any impact … people bring up the contaminated blood scandal. What can we argue? The experts, the authorities sometimes lack objectivity, reasoning. They are also political. So … I understand that people are asking questions (Dr. Amidane).

Along these lines, most GPs (14/19) clarified that their job was to inform patients rather than to encourage vaccination:

I explain [what the vaccine is] … but then it’s their responsibility! … *you told me it tired you out* … I do my work … I must inform them and then it’s absolutely not my problem. I tell them “if you believe the internet believe the internet, it’s your problem!” (Dr. Vincent).

Discussing and enforcing (notably mandatory) vaccination can be especially difficult for vaccine hesitant GPs, and may restrict their capacity for exercising professional judgement, so that they feel their expertise is undermined. This means that mandatory vaccination can be experienced by some GPs as coercive. For example, when referring to the fact that the mandatory pertussis vaccine currently also contains vaccines against diphtheria and tetanus, Dr. Moreau stated, “We have no choice! We have choices in theory but in practice we don’t. It creates consultations that are a little tense”. This tension places additional burdens on GPs who, at the interface of vaccine experts/policymakers, and patients, must bear the brunt of patient dissatisfaction when expert systems are perceived to fail:

There are lots of unanswered questions, it’s a bit awkward because when you vaccinate someone who’s questioning it, we understand … we always have the information late … Take Meningitec [the vaccine against meningitis C was withdrawn as a precautionary measure following concerns about particulate contamination] … there was a case of side effects and everyone learned about it in the press the next day, we received a letter a month later from the authorities … So you’re seen as ridiculous when you then offer Meningitec, which was my case the day before the event and the patient asked, “were you not aware?” so we’re always out of step … the information’s delayed, so I have no confidence in it … it’s very unpleasant for us … saying [to the patient], “don’t worry”. We always try to smooth things out but we know nothing (Dr. Bernard).

One could ask how GPs can do what they are expected by the authorities (use clinical expertise and reassure patients) when—it could be argued—they are effectively set up to fail; by not being informed about relevant and high-profile issues or changes to practice. As evidenced in the above quote, this undermines GPs and their practice.

To summarize, the authorities’ requirement for GPs to recommended certain vaccines and enforce acceptance of others clashes with the notion of patient choice which according to participants was so important to cultivating a trusting relationship between themselves and their patient. This threat to choice was also evident among GPs themselves who felt bound by the vaccine policies made by the authorities, and which they were not consulted on. Various factors including feeling undermined by these requirements sometimes made it difficult for GPs to effectively discuss vaccination with patients, leaving the latter to make important decisions on their own.

## Strengths and limitations

In a literature review on vaccine hesitancy, Larson, Clarke, et al., 2018 found a disconnect between the current vaccine hesitancy research and the wider health-related trust literature, with few studies exploring trust among HCPs, factors outside of the vaccination programme or asking about specific vaccines. The authors stated that the important concept of trustworthiness of the systems themselves was noticeably absent and recommended future research into further interactions between the various dimensions of trust and vaccination.

Our study was an in-depth exploration into, and analysis of the various tensions GPs face and some GPs’ distrust in health authorities and thus vaccination. This was achieved through an analysis of scandals related to the health care system and by drawing on the wider health-related trust literature, with a focus on “reluctant trust” and the reliance of social, informal sources of knowledge. The study therefore filled several research gaps mentioned above.

Through a telephone questionnaire asking vaccine hesitant GPs about specific vaccines for which coverage is suboptimal (adapted from the 2014 quantitative survey analysing GPs’ vaccine attitudes and practices (Verger et al., 2016)) and then by elucidating GPs’ answers to the telephone questionnaire through face-to-face semi-structured interviews, this study complemented and enhanced the results of the quantitative survey; participants had time to explain their answers and elaborate on their responses to the telephone questionnaire. The importance of this approach was shown by the fact that six participants changed at least one of their answers to the telephone questionnaire during the face-to-face interview, adding extra insight and nuance into their answers of the questionnaire (Shown in red in Appendix B).

The response rate during recruitment was low and a few interviews were relatively short. However, saturation was reached (with similar concerns being expressed among participants before the interviews were finished) and the aim of the study was not for results to be generalizable to all GPs, but to vaccine hesitant GPs in France and perhaps in other countries with a similar context and health care system. Additionally, as most potential participants could either not be contacted, or refused to participate before they knew that the study was about vaccination, it is unlikely that they did not participate due to not wanting to discuss vaccination. The low response rate and the fact that some interviews were relatively short, is also unsurprising given the demanding nature of this cohort’s occupation.

An important future study in this field could involve interviewing GPs who are not vaccine hesitant and to compare the findings with those of this study.

## Discussion

Larson, Clarke, et al., 2018 define trust as one party being in a vulnerable position, assuming the best interests and competence of the other (as well as parties linked to the other), in exchange for a reduction in decision complexity. Such trust is determined by a “web” of mutually interacting relationships between individuals and social systems (Meyer et al., 2008). In this context, and between HCPs and health care authorities specifically, we take the above definition further to argue that trust works both ways and so engenders an implicit contract. As well as a reduction in decision complexity, this should involve authorities providing HCPs with support and in turn, HCPs recommending vaccination. Our findings suggest that for some GPs in France, this implicit contract has been ruptured.

This is because the historical legacy of trust/distrust derived from interactions with official institutions influences generalized trust in society, and vaccine-related trust exists within the context of deeper, underlying trust in society at large. This means that the past actions of a health system and the perceived values that it holds, play a substantial role in trust in the vaccines that it recommends (Larson, Clarke, et al., 2018). This was evident in our study, where sentiments surrounding trust in the authorities were central to vaccination discourse.

Following the numerous health care scandals, lack of support from authorities and conflicting, insufficient, and perceived biased information; uncertainty and distrust, or (as referred to by Giddens (1991)), “reluctant trust” in the experts, health authorities and various vaccines ensued. Rather than base a decision on “rational choice”, reluctant trust combines reasoning (from past experience) and a leap of faith (Simmel, 1990). By consciously bracketing out issues and uncertainty that individuals cannot remove or resolve fully, but that also should not disrupt practice, uncertainties are neutralized creating an *illusion* that issues are favourably resolved (Dr. Bernard referred to “playing his role as a doctor” when discussing vaccination with patients). Reluctant trust therefore allows individuals to trust at least to an extent, making it easier to deal with and act on difficult advice or decisions (Giddens: ch.1), and in our study, make it easier for some vaccine hesitant GPs to practice. However, reluctant trust also engenders shouldering responsibility and blame if anything were to go wrong after vaccination. This has been found among parents when making vaccine decisions (Ward et al., 2017), and was evident in our study when supposed adverse events following vaccination of both Dr. Laurent and Dr. Marie’s patients left the GPs feeling “shaken up” and reluctant to encourage future vaccination.

Several other, less obvious issues can also arise with a lack of support from authorities and the resulting reluctant trust. The state of suspending doubt that is required to take a leap of faith is fragile and can be followed by a regression into a “suspension of trust” that can lead to existential anxiety (Giddens, 1990) (as evident in the existence of vaccine hesitant GPs). This can lead to feelings of confusion, alienation or even paralysis of action (Luhmann, 2000).

Additionally, a relationship where one has no choice but to place faith in someone or something is not so much a trusting relationship but one of dependency or compliance (Davies & Mannion, 1999). GPs in this study—while distrusting the health care authorities to an extent—depended on them for the legally binding guidelines that define their practice and that of all other GPs in the country. This means that the implicit contract between authorities and GPs may not only be ruptured but broken; instead of mutual understanding, GPs may simply be given instructions that they are expected to follow. This can inevitably lead to further negative consequences regarding their levels of trust in health authorities and vaccination.

In this study, the distrust in the healthcare authorities and a lack of support from them meant that following or adapting vaccination guidelines and dealing with uncertainties was often aided by GPs drawing on trusted personal, non-certified sources of expertise rather than official guidelines, which traversed boundaries between professional and lay fields of expertise and experiences (Eyal & Pok, 2015). Like the public, GPs are aware that experts disagree with each other, that science and technology often generate risks, and that, funded by a complex system of state and international organizations and private companies, the development and delivery of vaccines involves conflicting political and financial motives (Giddens, 1990). Today questioning science is not a sign of ignorance but is instead endorsed by highly educated individuals (Beck, 1992) (even if not always by “scientists” and health authorities), with the perception that the biggest risk is trusting blindly (Hobson-West, 2007). This has been aided by the availability and proliferation of a wide variety of information (especially today through social media platforms) endemic to late modern existence (Foucault, 1976). While one study in France found that only six percent of GPs trust vaccine information from the media (Collange et al., 2015), interestingly GPs in our study generally did not mention the media as an information source (apart from one who mentioned that people are misinformed or “too informed” due to the media reporting issues that are not scientifically correct). However, over a quarter said that they relied on information from the journal *Prescrire*; a non-profit association financed by subscribers (mainly GPs and pharmacists), having no grants, advertising, shareholders or sponsors (Prescrire, 2019). *Prescrire* is thus positioned as aiming to defend the interest of patients, sheltered from the influences of various lobbies (Meidani, 2018). Interestingly, although it does not publish any research articles, *Prescrire’s* English language version is indexed in Medline. Thus, in what has been referred to as the rise of the “knowledge society”, scientists are no longer held to be all-knowing, guiding figures (Grundmann, 2017). GPs’ are thus in a paradoxical position of being both health care representatives who are expected to implement vaccine guidelines and possess vaccine expertise, and citizens who are challenged by continued uncertainties about what information and advice to trust.

Similar to our study, in an ethnographic study examining the ways that GPs come to their individual and collective health care decisions, Gabbay and Le May (2004) found that clinicians rarely accessed and used explicit evidence from scientific research directly, but relied on what the authors termed “mindlines”, which are “collectively reinforced, internalised, tacit guidelines”. These were informed through a range of informal interactions in “communities of practice” and mainly consisted of their own and their colleagues’ experiences, and interactions with each other, trusted opinion leaders and patients. Human thought, explanations and judgements are thus not constructed by individuals, but in the “permanent dialogue” that people have with each other and with institutions (Joffe, 2003). This identifies courses of action and reduces complexity and uncertainty (Stehr & Grundmann, 2011), (Wilson, 2017).

In our study GPs’ reliance on trusted “informal” knowledge sources, which facilitated their adaption of clinical guidelines, often instead of relying on statistics and risks/benefit analyses, is reminiscent of public reliance on trusted personal information sources; indeed, some GPs drew on their own experiences of being parents in their interactions with patients. In a study on HPV vaccine communication in Sweden, Linden (2016) found that girls trusted vaccination information more if it was provided by somebody they knew (i.e., a school nurse). Linden argues that it is not vaccination information itself that causes trust or distrust in vaccination, but the people connected to the information. This is in line with Giddens’ (1990) argument of the necessity to anchor trust in face-to-face relationships.

It is also important to acknowledge that in neoliberal societies of deregulation, health care can resemble a marketplace, where patients choose their care, and so are perceived as “customers” (Mol, 2008). This approach has been especially evident in France since the introduction of *The law on the rights of the sick and the quality of the health system* in 2002, which claims to better meet the expectations of patients (Cardin, 2014). While this approach clearly has benefits regarding patient rights, it highlights the second tension identified though this study; there currently exists a health care ideal for “patient choice” and autonomy, which is coupled with GPs wanting to adapt to patient vaccine hesitancy in order to preserve positive relationships with them (especially in France where patients can choose their GP). This clashes with the desire of health care authorities for all patients and HCPs to adhere to vaccination policy and therefore make the “right choice” to vaccinate (Mol, 2008).

We must also consider the fact that in the case of childhood vaccination, decisions are often not taken by the patient but by their parent/guardian. This means that informed consent is not taken by the person who might be affected by any side-effects or complications from being vaccinated or not. This makes the decision-making process for both the parent/guardian and GP more complex (Hendrix et al., 2016), especially as vaccination is something a child normally does not want (it hurts, and it might be hard for them to foresee consequences regarding risks and protection).

The expected use of formulaic procedures in practice can also carry negative implications for physicians’ learning and thus can erode clinical autonomy (Rappolt, 1997), leading to GPs feeling excluded, especially if they are not supported or respected by the authorities that implement them. This in turn affects the quality of patient care (Smith et al., 2003) and patient choice (Rogers, 2002) because despite having an important role in determining health outcomes, strict official guidelines can overlook socio-cultural, political and economic dimensions of individual patients, as well as doctor and patient narratives (Lambert, 2006). Indeed, scientific discovery and knowledge may not always conform to what is traditionally viewed as “rational” (Kuhn, 1970). The overarching tension for GPs thus lies in how they balance the interests of the health authorities and their patients. Some may deal with these tensions by detaching themselves from their difficult position between experts and patients, through solely providing vaccine information, rather than seeking to reassure vaccine hesitant patients though discussion. This could not only come across as dismissive of vaccination but goes against what many patients want; clear guidance and support in making decisions. If this is not provided, patients can feel neglected and may disengage with vaccination discussions altogether (Wilson, 2017).

In conclusion, if the state’s role is not seen as legitimate and trustworthy with regards to vaccine recommendations, then not only patients distrust health interventions such as vaccination or certain vaccines, but HCPs can feel uncertain and unsupported. If this happens, they lack an acceptable, workable framework for engaging with patients and each-other (Brownlie & Howson, 2006), as well as official vaccination guidelines. This inevitably has negative consequences for trust in, and acceptance of vaccination.

In order to overcome some of these issues, GPs should be more involved in decisions around vaccination policy. Such an approach could take the form of working groups where HCPs, concerned publics, and social science academics are invited into the design process of vaccination campaigns. A similar approach has been implemented by a number of National Health Service (NHS) Trusts in the UK since September 2016 and has been viewed favourably by those who have been involved with it (Seale, 2016).

Discussion about, and incorporating a broader conception of vaccination in context would mean that one-way information is replaced with dialogue that appreciates and understands the social processes around vaccination concerns (Poltorak et al., 2005), and would acknowledge that these processes are valid (Kukla, 2005). This approach could foster relationships of collaboration, address circumstances that may hinder GPs’ autonomy, and help health care authorities to build trusting relationships with GPs.

In-depth analyses of vaccine hesitancy among HCPs globally could further uncover problematic actions taken by health authorities, and where communication and support for HCPs surrounding vaccination may be lacking. There is also a need for further research exploring how HCPs rely on personal sources of guidance rather than official guidelines, and how this affects their vaccine discussions with patients, as this study showed that the effects on patients of HCPs relying on personal sources of guidance are mixed.

Ultimately, by shifting the burden of distrust from the individual or community, onto the trustworthiness of institutions, the genuine drivers of trust and distrust may become clear. In highlighting the perception and expectation gulfs between HCPs and health authorities, coupled with tailored suggestions of how to overcome them, ruptured or broken implicit contracts between both parties could gradually be re-built, contributing to increased trust in health care authorities and thus vaccination.

## Notes

1. See Appendix A for a map of study sites.

2. This is the most recent available data.

3. National immunization schedule.

## Appendix A.

Map of recruitment sites.

## Appendix B.

**Table:** Responses to the telephone survey with answers changed during the face-to-face interview highlighted

GP	Questions																
	Regarding vaccination, in your daily practice are you … ?	Do you offer MMR to non-immunized adolescents and young adults?	Do you offer Meningococcus C for 12-month-old infants?	Do you offer Meningococcus C booster for 2 to 24 year-olds?	Do you offer HPV for 11–14 year-old girls?	Do you offer the hepatitis B booster for adolescents?	Do you offer the seasonal flu vaccine for adults under the age of 65?	The hepatitis B vaccine causes multiple sclerosis	The HPV vaccine causes multiple sclerosis	Adjuvants cause long-term complications	Today some recommended vaccines are unnecessary	Today children are vaccinated against too many diseases	Do you trust the MoH to give you reliable vaccination information?	Do you trust health agencies to give you reliable vaccination information?	In terms of vaccination, health authorities are influenced by the pharmaceutical industry	In terms of vaccination, do you rely on your own judgement rather than official recommendations?	Have any of your patients experienced a serious health problem that was potentially linked to a vaccination?
**Dr. Martin**	Mostly favourable	Sometimes	Never	Never	Sometimes	Never	Sometimes	Likely	Not at all likely	Yes	Agree	Agree	Distrust	Distrust	Mostly agree	Agree	No
**Dr. Bernard**	Very favourable	Systematically	Systematically	Systematically	Never	Systematically	Systematically	Not at all likely	Don’t know	No	Disagree	Disagree	Distrust	Mostly trust	Mostly agree	Mostly agree	Yes
**Dr. Dubois**	Very favourable	Systematically	Sometimes	Never	Systematically	Sometimes	Systematically	Not at all likely	Not at all likely	Unlikely	Disagree	Disagree	Mostly trust	Mostly trust	Don’t know	Disagree	No
**Dr. Thomas**	Very favourable	Often	Systematically (changed to Often)	Often	Often	Often	Often	Not at all likely	Not at all likely	No	Disagree	Disagree	Mostly trust	Mostly trust	Mostly agree	Disagree	No
**Dr. Durand**	Very favourable	Often	Often	Never	Never	Systematically	Systematically	Not at all likely	Not at all likely	Don’t know	Disagree	Disagree	Mostly trust	Mostly trust	Mostly agree	Mostly disagree	No
**Dr. Moreau**	Very favourable	Often	Sometimes	Often	Systematically	Systematically	Not at all likely	Not at all likely	Unlikely	Mostly disagree	Mostly disagree	Mostly disagree	Distrust	Mostly trust	Mostly agree	Disagree	Yes
**Dr. Laurent**	Very favourable	Systematically	Sometimes	Sometimes	Often	Sometimes	Sometimes	Don’t know	Don’t know	Unlikely	Disagree	Disagree	Distrust	Mostly trust	Mostly agree	Mostly agree	Don’t know
**Dr. Michel**	Very favourable	Often	Systematically	Often	Systematically	Systematically	Systematically	Not at all likely	Not at all likely	Don’t know	Disagree	Disagree	Mostly trust	Trust	Mostly agree (changed chd to Disagree)	Mostly agree	No
**Dr. Garcia**	Very favourable	Sometimes	Sometimes	Sometimes	Sometimes	Sometimes	Often	Don’t know	Don’t know	Unlikely	Don’t know	Don’t know	Distrust	Mostly trust	Agree	Mostly agree	No
**Dr. Amidane**	Mostly favourable	Often	Sometimes	Sometimes	Systematically	Never	Systematically	Don’t know	Don’t know	Unlikely	Agree	Mostly agree (changed to Disagree)	Distrust	Mostly trust	Disagree	Agree	No
**Dr. Roux**	Very favourable	Often	Often (changed to Systematically)	Sometimes (chd to Systematically)	Sometimes	Sometimes	Not at all likely	Not at all likely	Unlikely	Mostly disagree	Disagree	Disagree	Mostly trust	Mostly trust	Mostly disagree	Mostly disagree	No
**Dr. Vincent**	Very favourable	Often	Often	Often	Often	Often	Sometimes	Not at all likely	Not at all likely	Unlikely	Mostly disagree	Mostly disagree	Don’t know	Mostly trust	Don’t know	Mostly disagree	No
**Dr. Fournier**	Very favourable	Systematically	Systematically	Often	Often	Sometimes	Often	Not at all likely	Not at all likely	Unlikely	Mostly agree	Disagree	Mostly trust	Mostly trust	Don’t know	Mostly disagree	No
**Dr. Morel**	Mostly favourable	Often	Systematically	Systematically	Systematically	Systematically	Systematically	Not at all likely	Not at all likely	Unlikely	Mostly disagree	Disagree	Mostly trust	Mostly trust	Mostly disagree (changed to Don’t know)	Disagree	No
**Dr. Andre**	Very favourable	Sometimes	Systematically	Sometimes (changed to Often)	Often (changed to systematically)	Sometimes	Often	Not at all likely	Don’t know	Unlikely	Disagree	Mostly disagree	Distrust	Mostly trust	Mostly agree	Mostly disagree	No
**Dr. Duval**	Very favourable	Often	Sometimes	Sometimes	Often	Systematically	Systematically	Don’t know	Don’t know	Don’t know	Disagree	Disagree	Mostly trust	Mostly trust	Don’t know	Mostly agree	No
**Dr. Lucas**	Mostly favourable	Sometimes	Systematically	Often	Often	Sometimes	Systematically	Not at all likely	Don’t know	Likely	Mostly disagree	Disagree	Mostly trust	Trust	Mostly agree	Disagree	No
**Dr. Marie**	Mostly favourable	Never	Sometimes	Never	Often	Sometimes	Often	Not at all likely	Not at all likely	Don’t know	Mostly agree	Mostly agree	Mostly trust	Mostly trust	Don’t know	Mostly agree	Yes
**Dr. Rey**	Very favourable	Systematically	Often (changed to systematically)	Systematically	Systematically	Systematically	Systematically	Not at all likely	Not at all likely	No	Disagree	Disagree	Mostly trust	Mostly trust (changed to Trust)	Disagree	Disagree	No
